# Differential modification of perceptual thresholds by prolonged near threshold motion in healthy adults and after peripheral lesions

**DOI:** 10.3389/fneur.2025.1542496

**Published:** 2025-03-12

**Authors:** Andrew R. Wagner, Soroush G. Sadeghi, Daniel M. Merfeld

**Affiliations:** ^1^Department of Physical Therapy, School of Pharmacy and Health Professions, Creighton University, Omaha, NE, United States; ^2^Department of Otolaryngology-Head and Neck Surgery, Johns Hopkins University, Baltimore, MD, United States; ^3^Department of Otolaryngology-Head and Neck Surgery, Ohio State University, Columbus, OH, United States; ^4^Department of Biomedical Engineering, Ohio State University, Columbus, OH, United States; ^5^Health and Rehabilitation Sciences, Ohio State University, Columbus, OH, United States; ^6^Speech and Hearing Sciences, Ohio State University, Columbus, OH, United States

**Keywords:** vestibular, perceptual threshold, perception, plasticity, vestibular hypofunction

## Abstract

**Purpose/hypothesis:**

Homeostatic plasticity is an innate self-regulatory process that functions to stabilize neural excitability in response to sensory perturbations. The purpose of this study was to investigate homeostatic plasticity in vestibular perceptual responses by measuring changes in vestibular perceptual thresholds after exposure to passive whole-body self-motion stimuli (vestibular conditioning). We hypothesized that small amplitude stimuli (i.e., subthreshold conditioning) would cause a decrease in thresholds, whereas large amplitude stimuli (i.e., suprathreshold conditioning) would cause an increase in thresholds.

**Methods:**

One-Hz yaw rotation vestibular perceptual thresholds were measured before and immediately after 20-min blocks of passive whole-body motion (i.e., conditioning) in a cohort of 12 healthy adults (27 ± 8.19 years; 10 female). The conditioning stimuli consisted of 1 Hz sinusoidal motions and included (a) subthreshold yaw rotations with a peak velocity equal to 57.4% of the baseline threshold (T_0.57x_), (b) suprathreshold yaw rotations with a peak velocity equal to 200% of the baseline threshold (T_2x_), or (c) a sham stimulus consisting of 0.1 mm/s interaural translations (T_Sham_). A subset of the group returned to complete an additional subthreshold yaw rotation condition with a peak velocity equal to 20% of the baseline threshold (T_0.2x_). A cohort of 5 individuals (1 female) with chronic unilateral vestibular hypofunction participated in the T_0.57x_ subthreshold conditioning stimulus.

**Results:**

Yaw rotation thresholds were significantly increased after suprathreshold conditioning (1.36 ± 0.75°/s, *p* = 0.004), increasing an average of 31.75% relative to baseline (1.05 ± 0.52°/s). However, counter to our hypothesis, yaw rotation thresholds were not significantly lowered in our healthy adult population after either of the two subthreshold conditioning tasks (T_0.57x_: 1.11 ± 0.62°/s, *p* = 0.61; T_0.2x_: 1.20 ± 0.69°/s, *p* = 0.385). Yet, four out of the five participants with chronic unilateral vestibular hypofunction displayed an improvement in perceptual thresholds (Range of 10.32–29.14%) following the T_0.57x_ subthreshold conditioning task.

**Conclusion:**

These data suggest (1) that 20 min periods of passive whole-body motion are sufficient to modify vestibular perception and (2) that the impact of subthreshold conditioning on perceptual thresholds may depend on the baseline integrity of the vestibular system.

## Introduction

Homeostatic plasticity is a type of synaptic plasticity that functions to stabilize the excitability of neurons in response to a sustained change in the neural firing rate (1). This process scales neuronal excitability in the direction opposite to the sensory perturbation, increasing excitability in response to lowered firing rates and decreasing excitability in response to increased firing rates (2, 3). Although early studies have focused primarily on the visual cortex and spinal motor circuits (4–7), recently it has been shown that similar self-regulatory mechanisms may also influence the sensitivity of the vestibular system to head motion stimuli (8–11).

Plasticity in the central vestibular pathways has been well characterized under abnormal conditions such as after unilateral vestibular deafferentation (12, 13). Following a unilateral loss of vestibular firing, vestibular nuclei neurons shut down initially, but regain their resting discharge and increase their sensitivity to inputs over a few days (12, 14–17), resulting in normalization of VOR responses over time (18–21). Under these abnormal conditions, it has also been shown that VOR responses could be further improved by rotations that stimulate the weaker side and inhibit the stronger side (22–26). The latter functions in a homeostatic way to rebalance the two sides. However, such unidirectional ipsilesional stimuli would result in a different homeostatic response under normal conditions, when vestibular responses are symmetric and/or intact. As a result, there is a need to better understand the mechanisms responsible for symmetrically increasing or decreasing vestibular responses. Such mechanisms may be leveraged for specific therapeutic purposes, such as (a) increasing vestibular responsiveness in individuals with vestibular dysfunction (e.g., aminoglycoside exposure), (b) optimizing performance in individuals without vestibular pathology (e.g., pilots and astronauts), or (c) reducing abnormal hypersensitivities to motion (e.g., Persistent Perceptual Postural Dizziness and motion sickness).

Recently, brief exposures to different amplitudes of bidirectional vestibular stimulation have been shown to modify behavioral and physiological vestibular responses in a manner consistent with homeostatic plasticity. In an intact *Xenopus laevis* sample, Dietrich and Straka identified a bidirectional modulatory effect of sinusoidal rotations on neuronal firing rates within the vestibulo-ocular reflex (VOR) pathway (8). Specifically, they found that conditioning to low velocity rotations at 0.5 Hz increased response sensitivities of oculomotor nerves to 0.5 Hz rotations whereas higher velocity rotations reduced their sensitivities. Importantly, these changes occurred independent of visual feedback, and were constrained only to the neurons within the pathway coinciding with the plane of rotation (8). The effects of bidirectional whole-body motion have also been explored in humans with intact vestibular sensation. Consistent with the response seen by Dietrich and Straka, separate studies by Keywan and colleagues found reductions in vestibular translation thresholds of between 28.8% (9) and 39% (10) at 1 Hz, after a single 20-min block of low velocity (i.e., subthreshold) 1 Hz interaural sinusoidal translation. Notably, this effect lasted for less than 20 min and did not translate to rotational responses. In another study, Fitzpatrick and Watson (11) found that a single 10-min block of large amplitude stochastic whole body yaw rotations (0.5–2.5 Hz, 100 deg/s) led to a 248% decrease in perceptual sensitivity to a step rotation and a 50% decrease in the postural response to a galvanic vestibular step pulse (11). While these effects recovered within an hour after conditioning, the galvanic response did not return to control values. Collectively, these studies demonstrate that motion perturbations (i.e., low or high levels of vestibular stimulation) provide a suitable paradigm for inducing changes in vestibular sensitivity.

The present pilot study was designed to determine if this presumed capacity to bidirectionally modify vestibular sensitivity could be elicited by varying the intensity of a sinusoidal yaw rotation stimulus relative to an individualized baseline level of motion sensitivity. In addition, we aimed to determine the feasibility of utilizing subthreshold conditioning to augment central compensation in a cohort of individuals with peripheral vestibular lesions. Based upon the bidirectional modulation of vestibular responses identified by Dietrich and Straka, we hypothesized that subthreshold yaw rotations would result in increased sensitivity to yaw rotation, while suprathreshold rotations would lead to reduced sensitivity. The rationale for this hypothesis being that periods of subthreshold vestibular stimulation may elicit plastic changes similar to those experienced under conditions of vestibular damage, where neurons in the vestibular nuclei compensate for the reduction in afferent input by increasing their sensitivity to motion stimuli through homeostatic mechanisms. Suprathreshold stimulation, as described above, may act through similar mechanisms, yielding a reduction in the sensitivity of the involved neurons.

## Methods

Twelve asymptomatic adult participants were recruited from the Ohio State University campus and served as healthy controls (HC) for the study. We recruited individuals who reported no history of vestibular, or other neurologic disorders. Preservation of lateral canal function was confirmed at baseline using video head impulse test (vHIT) in the horizontal plane. A bilateral vestibulo-ocular reflex (VOR) gain >0.8 was considered normal. The yaw VOR gain was calculated using published methods by taking the median ratio between eye velocity and head velocity across the 30 msec window preceding peak head velocity (27). Three separate conditioning blocks—subthreshold, suprathreshold, and sham—were tested on separate days in a randomized, counter-balanced order. In 9/12 of these participants a fourth condition was also tested to investigate an additional subthreshold conditioning stimulus.

Individuals with unliteral vestibular hypofunction (UVH) were recruited from the Ohio State Wexner Medical Center. Subjects had a history of peripheral vestibular hypofunction in their electronic medical record, which was confirmed by a unilateral vHIT gain <0.8. The individuals with UVH only participated in the subthreshold conditioning task. In each condition participants were blinded to the nature of the motion and were informed only that they “may or may not move” during the conditioning block. Each participant provided informed consent, and the study protocol was approved by The Ohio State University Institutional Review Board.

### Vestibular perceptual thresholds

Before and after each 20 min conditioning block, each participant completed a self-motion direction recognition task to determine a 1 Hz yaw rotation vestibular perceptual threshold (28). Individuals were asked to judge the direction of a 1 Hz (1 s per cycle of acceleration) yaw rotation stimulus consisting of a single cycle of sinusoidal acceleration [see Figure 2 in (29)] while sitting in a dark room, with insert earphones playing ~60 dB SPL (sound pressure level) white noise. Participants provided a response only after the stimulus had ended. Using methods that we have previously described (29–31), the protocol began with a 2-down/1-up (2D1U) initial staircase until the first incorrect response, and then proceeded to use a 4-down/1-up (4D1U) staircase for the remainder of the trials. Each step size in the staircase was selected using standard Parameter Estimation by Sequential Testing (PEST) rules (32). After each response was given, the chair remained stationary for ~3 s to avoid motion aftereffects (33, 34). To maintain alertness and attention, participants were notified when they reached between 50 and 75 trials, but otherwise were not given feedback regarding the accuracy of their responses. The HC cohort completed 150 trials per test session, whereas the UVH cohort completed 100 trials per test session.

The stimulus values, quantified as the peak angular velocity, and subject responses (right or left rotation) were fit to a Gaussian cumulative distribution function (CDF), and the threshold parameter was estimated using a bias-reduced generalized linear model with a probit link function (28, 35). This yielded the 1σ (“one-sigma”) threshold, which describes the width of the CDF, and represents the magnitude of a 1 Hz yaw rotation stimulus that a participant would be expected to accurately perceive on 84.1% of trials (28). In addition, we determined a bias parameter (*μ*) which describes the displacement of the CDF along the abscissa (28, 35). The bias parameter indicates asymmetry in the processing of self-motion cues arising from the bidirectional nature of the vestibular response to yaw rotation. While fitting the data, attentional lapses (i.e., responses given independent of the stimulus) were identified and removed using a published lapse identification algorithm (36). The lapse identification algorithm identifies potential outliers, i.e., trials likely to significantly change the threshold estimate, using a standard delete-one jackknife approach (36).

The precision of the perceptual threshold parameter depends on the sampling scheme used and the number of trials collected. A simulation-based study found that 150 trials of a symmetric 4D/1 U staircase yielded a 1σ threshold with a standard deviation of 13.2% (37). Since the standard deviation of thresholds has been shown to be approximately proportional to the inverse of the square root of the sample size, we pooled the perceptual responses from each visit’s baseline assessment to improve the precision of baseline threshold estimates (35, 37) and fit these data with a single psychometric function. Previous data in subthreshold conditioning suggest transient effects in healthy adults lasting <1 h (9) and thresholds estimated using similar methodologies to our own, have previously been shown to be stable between days of testing (38). Since the duration of any potential effects of conditioning are unknown for individuals with peripheral hypofunction, in accordance prior studies of VOR adaptation (39) additional baseline assessments were delayed 1-week in the UVH cohort.

### Vestibular conditioning

**Figure 1:**
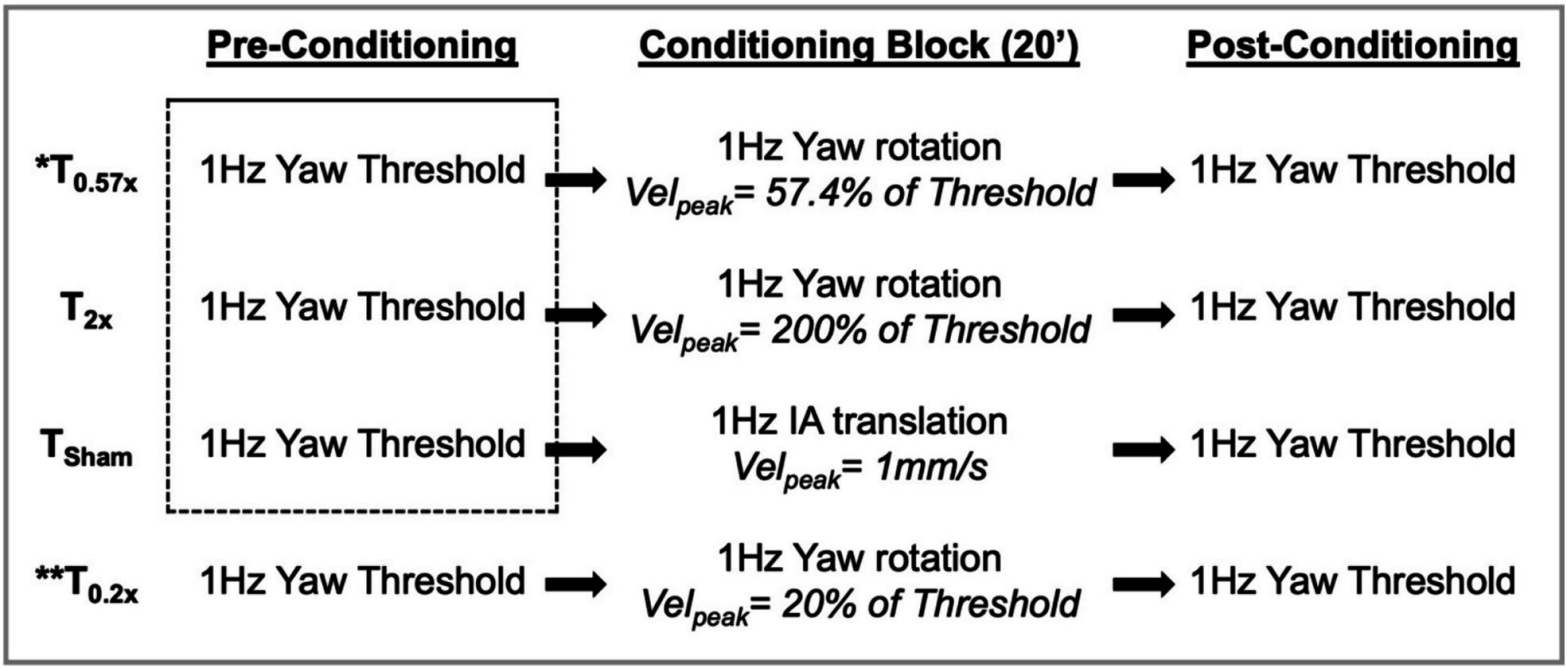
The experimental design is shown, with each of the 4 days of testing illustrated by one of the four rows. Stimuli used in the conditioning block were determined based upon the pre-conditioning threshold measured immediately prior. Baseline thresholds were estimated by pooling trials from the first three pre-conditioning threshold assessments (dashed box) and were compared to the post-conditioning thresholds in the statistical analysis. IA, interaural; Vel_peak_, peak velocity. * also completed by 5 participants with unilateral peripheral vestibular hypofunction; ** completed by 9 out of the 12 participants.

Three different conditioning stimuli (T_0.57x_, T_2x_, and T_Sham_) were tested in each of the 12 HC participants in a randomized and counterbalanced order. Nine out of these 12 participants were able to return for a T_0.2x_ condition that was added after data collection had begun. The UVH cohort participated only in T_0.57x_ conditioning. Conditioning stimuli consisted of 20-min blocks of passive 1 Hz sinusoidal whole-body motion; a single condition was tested at each visit to the lab (Figure 1). Each motion profile was completed with the participant seated in a dark room, on a 6-DoF motion platform, with the head stabilized using a motorcycle helmet, and with insert headphones playing an audiobook or music of their choosing. After each conditioning block, the room lights remained off and the subject remained in the chair prior to the reassessment of 1 Hz yaw rotation thresholds.

The three stimulus blocks – T_0.57x_, T_0.2x_, and T_2x_ – tested the effect of sinusoidal motion with a peak velocity equal to 57%, 20%, or 200% of each individual’s baseline thresholds, respectively. T_0.57x_ was chosen to match a previous study that investigated the effects of subthreshold motion on the perception of interaural translation cues (9, 10). In this prior study, a 0.82σ threshold value was reported, rather than the 1σ threshold used here; a stimulus with a peak velocity of 57.4% of a 1σ threshold is equivalent to a stimulus set to 70% of a 0.82σ threshold (28). The T_0.2x_ condition was chosen based upon a prior animal study which found a modulatory effect of low amplitude yaw rotations performed at 20% of the reference motion condition (8). Unlike T_0.57x_ and T_0.2x_, T_2x_ tested the effects of a suprathreshold yaw rotation stimulus that could readily perceived by the participants. In addition to the three yaw rotation stimuli, a sham condition (T_Sham_) was also tested; an imperceptible (1 Hz, 1 mm/s peak velocity), interaural translation of the motion platform was used to provide subtle vibratory and auditory cues similar to those experienced during subthreshold motion, while minimizing stimulation of yaw rotation sensitive vestibular neurons.

### Data analysis

A linear mixed effect model was used to determine if the post-conditioning yaw rotation thresholds (T_0.57x_, T_Sham_, and T_2x_) significantly differed from baseline in the healthy control cohort (*N* = 12, significant at alpha <0.05). In each model, a subject identifier was included as a random effect term to adjust for inter-individual differences. The coefficients (*β*) represent the estimated difference between post-conditioning thresholds and baseline thresholds. To determine if the estimated differences after T_2x_ and T_0.57x_ conditioning were greater than differences after the sham, a linear combination of coefficients was performed following the mixed effect model (significant at alpha <0.025). To more directly test the *a priori* hypothesis of direction specific changes, we followed the primary analysis with paired one-tailed t-tests to compare post-conditioning thresholds to baseline. After normalizing the absolute value of the bias parameter by the threshold [abs(*μ*)/*σ*], identical methods to those described above were used to compare the normalized bias between the baseline assessment and each of the post-conditioning assessments. Each analysis was also repeated after adding results from the T_0.2x_ condition in the cohort of 9 participants who returned to complete the fourth day of testing. Spearman’s rho (*r_s_*) correlation coefficients were used to describe the association between baseline thresholds and the percentage of change after conditioning [(baseline-post)/baseline].

## Results

### Baseline thresholds and conditioning velocities

We tested a total of 12 healthy controls (HC) (27 ± 8.19 years old, 10 female) and a total of 5 adults with a history of unilateral peripheral vestibular hypofunction (1 female). None of the subjects in the HC cohort reported a history of vestibular pathology and they had a yaw VOR gain of at least 0.8 for right and left vHIT. In this group, 1 Hz yaw rotation thresholds measured prior to each of the conditioning blocks were not significantly different at each of the 3 visits to the laboratory (*F*(2,22.00) = 0.76, *p* = 0.48). Since baseline thresholds were not significantly different between visits, and since yaw rotation thresholds have been shown to be consistent across days of testing (38), we pooled the individual baseline responses from the 3 days of testing (150 trials each day) to improve the precision of the estimated baseline yaw rotation thresholds. After removing attentional lapses, this yielded an average of 449.58 ± 0.67 baseline trials (range of 448–450) per participant. Baseline thresholds (calculated from the pooled baseline trials) had an average value of 1.06°/s ± 0.52 (Table 1). We found similar baseline values of 1.08°/s ± 0.51 when thresholds were determined from the median of the three individual 150 trial pre-conditioning sessions.

**Table 1:** Baseline VOR gains and thresholds as well as post training thresholds are shown for all subjects.

	Diagnosis	Right vHIT gain	Left vHIT gain	Baseline threshold	Post T_0.57x_ threshold	Post T_2x_ threshold	Post T_sham_ threshold	Post T_0.2x_ threshold
*Healthy Control Cohort (N = 12)*								
	—	1.09 (0.07)	1.05 (0.08)	1.06°/s (0.52)	1.11°/s (0.62)	1.36°/s (0.75)	1.11°/s (0.53)	1.20°/s (0.69)
*UVH Cohort (N = 5)*								
UVH-1	R VNS	0.27	1.04	1.15°/s	0.93°/s			
UVH-2	R VNS	0.20	0.75	1.75°/s	1.38°/s			
UVH-3	L Lab.	0.74	0.16	2.54°/s	4.18°/s			
UVH-4	R Lab.	0.27	0.96	1.95°/s	1.75°/s			
UVH-5	R VN	0.13	0.85	1.78°/s	1.26°/s			

T_0.57x_, 57.4% of threshold; T_2x_, 200% of threshold; T_Sham_, 1 mm/s peak velocity 1 Hz interaural translations; T_0.2x_, 20% of threshold. The T_0.2x_ condition indicates data from 9 of the 12 participants. UVH, unilateral vestibular hypofunction; VNS, vestibular nerve section; Lab, labyrinthectomy; VN, vestibular neuritis. Each test condition was completed on a separate day.

Subjects in the UVH group had a history of at least 1 year. Four subjects had a history of right sided UVH, and one had a history of left sided UVH. Lateral canal vHIT gains were < 0.3 for the affected side for each participant (Table 1). Four of the five individuals in the cohort completed a total of 100 baseline trials prior to the T_0.57x_ conditioning block and returned 1–2 weeks later to complete an additional 200 baseline trials. Previous data in subthreshold conditioning suggest transient effects in healthy adults for <1 h (9), however in accordance with VOR adaptation studies, we opted to act conservatively and used a 1 week interval to reduce the potential for retention (39). The fifth individual completed all 300 trials in three separate blocks of 100 trials prior to the T_0.57x_ conditioning. Similar to the HC group, baseline thresholds were calculated by pooling responses from three testing sessions. At baseline, the UVH cohort (1.83 ± 0.50°/s vs. 1.06 ± 0.52°/s) demonstrated yaw rotation thresholds that were significantly elevated compared to the HC cohort (*t*(15)=2.84, *p* = 0.0125).

Personalized conditioning stimuli were calculated for each individual using the 1 Hz yaw rotation threshold measured at the onset of that day’s visit. In the HC cohort, T_0.57x_ conditioning stimuli were between 0.29 and 1.28 deg/s peak velocity (0.598 ± 0.31 deg/s), T_2x_ stimuli were between 1.069 and 5.35 deg/s (2.14 ± 1.20 deg/s), and T_0.2x_ stimuli were between 0.096 and 0.39 deg/s (0.23 ± 0.13 deg/s) (Table 2). In the UVH cohort, the T_0.57x_ stimuli were larger due to the increased baseline thresholds, with values between 0.75 deg/s and 1.56 deg/s (Table 2).

**Table 2:** The peak velocity (in units of degrees per second) of the conditioning stimuli used in each of the three conditions are shown for each individual.

	T_0.57x_ Conditioning		T_2x_ Conditioning		T_0.2x_ Conditioning	
	*Vel (Acc)*	% change after	*Vel (Acc)*	% change after	*Vel (Acc)*	% change after
*Healthy Control Cohort*						
HC 1	0.35 (1.10)	24.444	1.201 (3.77)	22.05	0.11 (0.35)	−18.428
HC 2	0.62 (1.95)	−12.735	1.408 (4.42)	−20.82	0.13 (0.40)	38.486
HC 3	0.42 (1.31)	14.575	1.651 (5.19)	49.92	0.19 (0.60)	−5.128
HC 4	0.31 (0.97)	−8.866	1.317 (4.14)	63.33	–	–
HC 5	0.32 (1.01)	8.930	1.069 (3.36)	75.04	0.11 (0.33)	−9.156
HC 6	0.29 (0.92)	−15.011	1.748 (5.49)	41.26	0.096 (0.30)	−33.043
HC 7	1.04 (3.27)	−6.565	3.30 (10.36)	49.36	0.39 (1.23)	18.123
HC 8	0.59 (1.85)	−1.418	1.91 (5.99)	7.82	–	–
HC 9	0.76 (2.40)	−10.625	2.66 (8.36)	−24.64	–	–
HC 10	0.67 (2.11)	64.385	1.83 (5.76)	100.18	0.28 (0.89)	34.475
HC 11	1.28 (4.02)	15.616	5.35 (16.81)	25.04	0.38 (1.19)	−2.969
HC 12	0.52 (1.64)	−17.597	2.28 (7.16)	−7.52	0.37 (1.16)	37.105
Median [IQR]	0.55 (1.74) [0.34, 0.72]	−3.99 [−12.21, 14.58]	1.79 (5.63) [1.36, 2.47]	33.15 [−3.68, 63.33]	0.19 (0.60) [0.11, 0.37]	−2.97 [−13.79, 36.45]
*UVH Cohort*						
UVH–1	0.68 (2.13)	−18.93	–	–	–	–
UVH–2	0.75 (2.36)	−20.72	–	–	–	–
UVH–3	1.56 (4.90)	32.78	–	–	–	–
UVH–4	0.85 (2.68)	−10.32	–	–	–	–
UVH–5	0.87 (2.74)	−29.14	–	–	–	–
Median [IQR]	0.85 (2.68) [0.75, 0.87]	−18.92 [−20.72, −10.32]	–	–	–	–

The conditioning stimuli represented cycles of sinusoidal acceleration, with the peak acceleration (shown parenthetically in units of degrees per second squared) being defined as V_peak_ * *π* * *f*, for the frequency (*f*) of 1 Hz.

### Effect of T_2x_ suprathreshold conditioning in healthy controls

**Figure 2:**
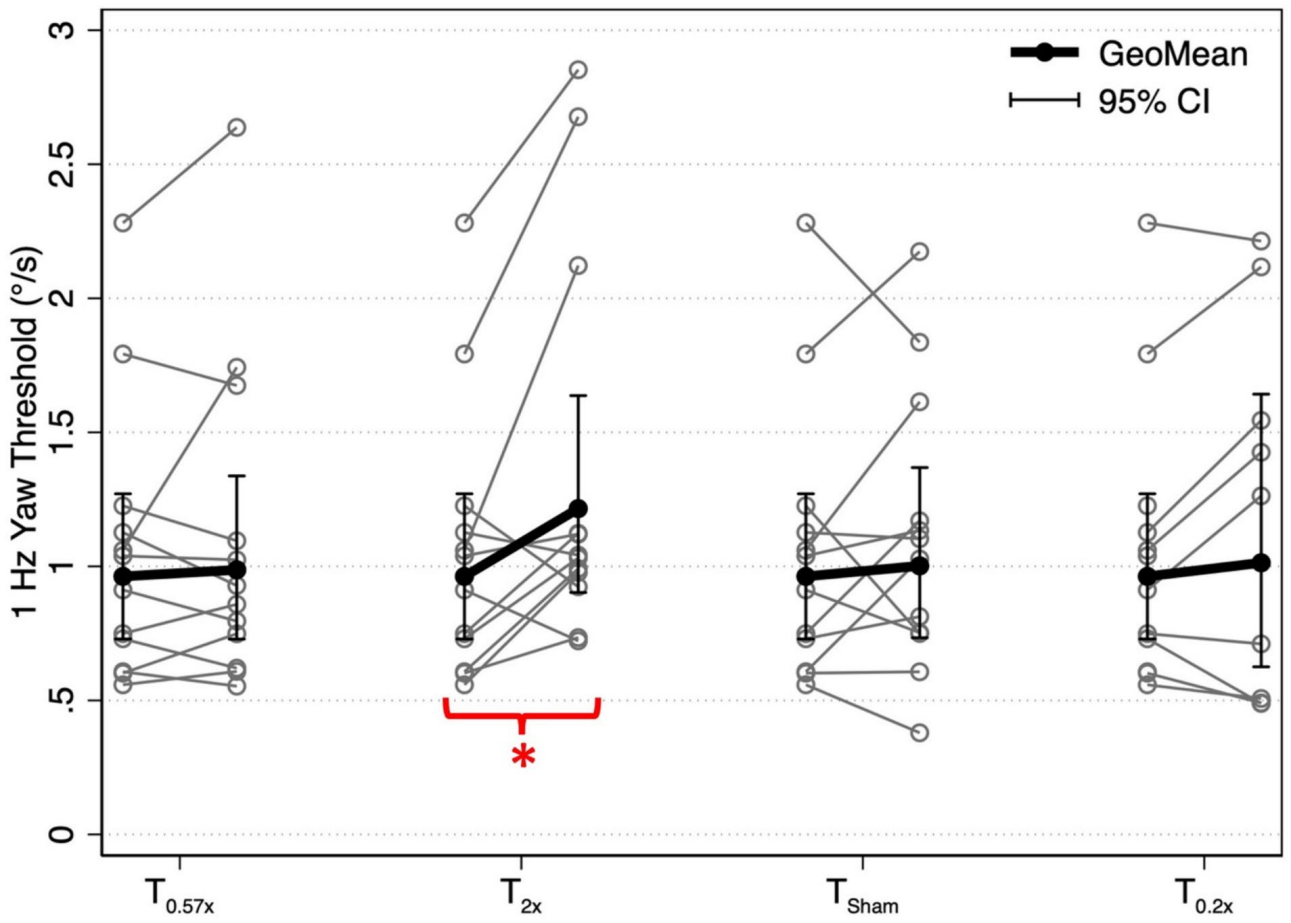
1 Hz yaw rotation perceptual thresholds are shown for each post-conditioning block relative to baseline yaw perceptual thresholds. Solid black lines show the geometric mean of yaw rotation thresholds, with error bars representing the 95% confidence intervals. Geometric means (GeoMean) are shown secondary to the log normal distribution of perceptual thresholds. All statistical analyses were performed on untransformed data and the results were unchanged when repeated after log transformation. T_0.57x_, 57.4% of yaw threshold; T_2x_, 200% of yaw threshold; T_Sham_, 1 mm/s peak velocity 1 Hz interaural translations; T_0.2x_, 20% of yaw threshold. The T_0.2x_ condition was completed by 9 of the 12 participants. * indicates a significant difference from baseline (*p* < 0.05).

**Figure 3:**
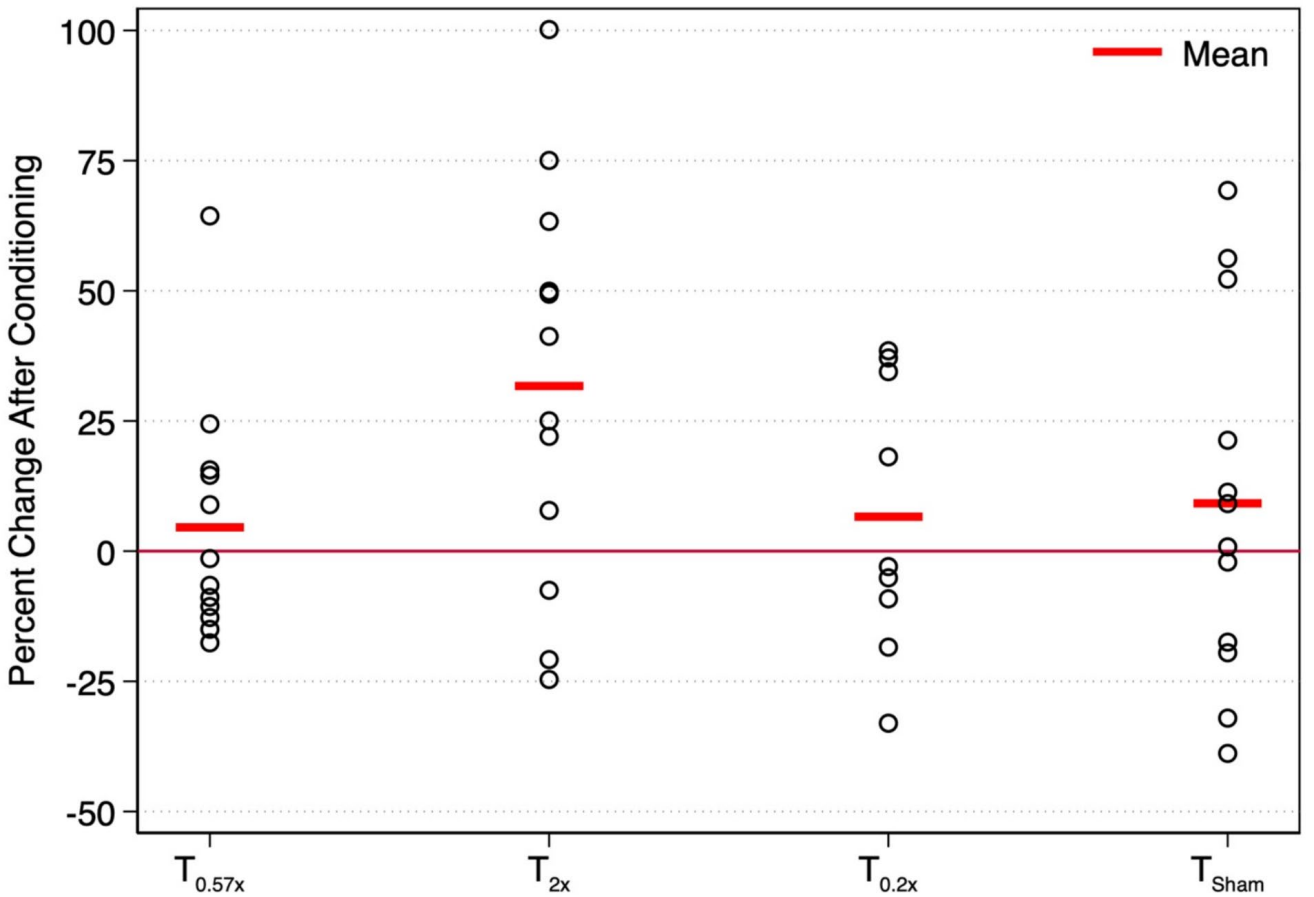
The percent change in yaw rotation thresholds after each conditioning task is shown for each of the 12 healthy control participants. Mean changes in thresholds across participants are indicated by horizontal red bars. T_0.57x_, 57.4% of threshold; T_2x_, 200% of threshold; T_Sham_, 1 mm/s peak velocity 1 Hz interaural translations; T_0.2x_, 20% of threshold.

**Figure 4:**
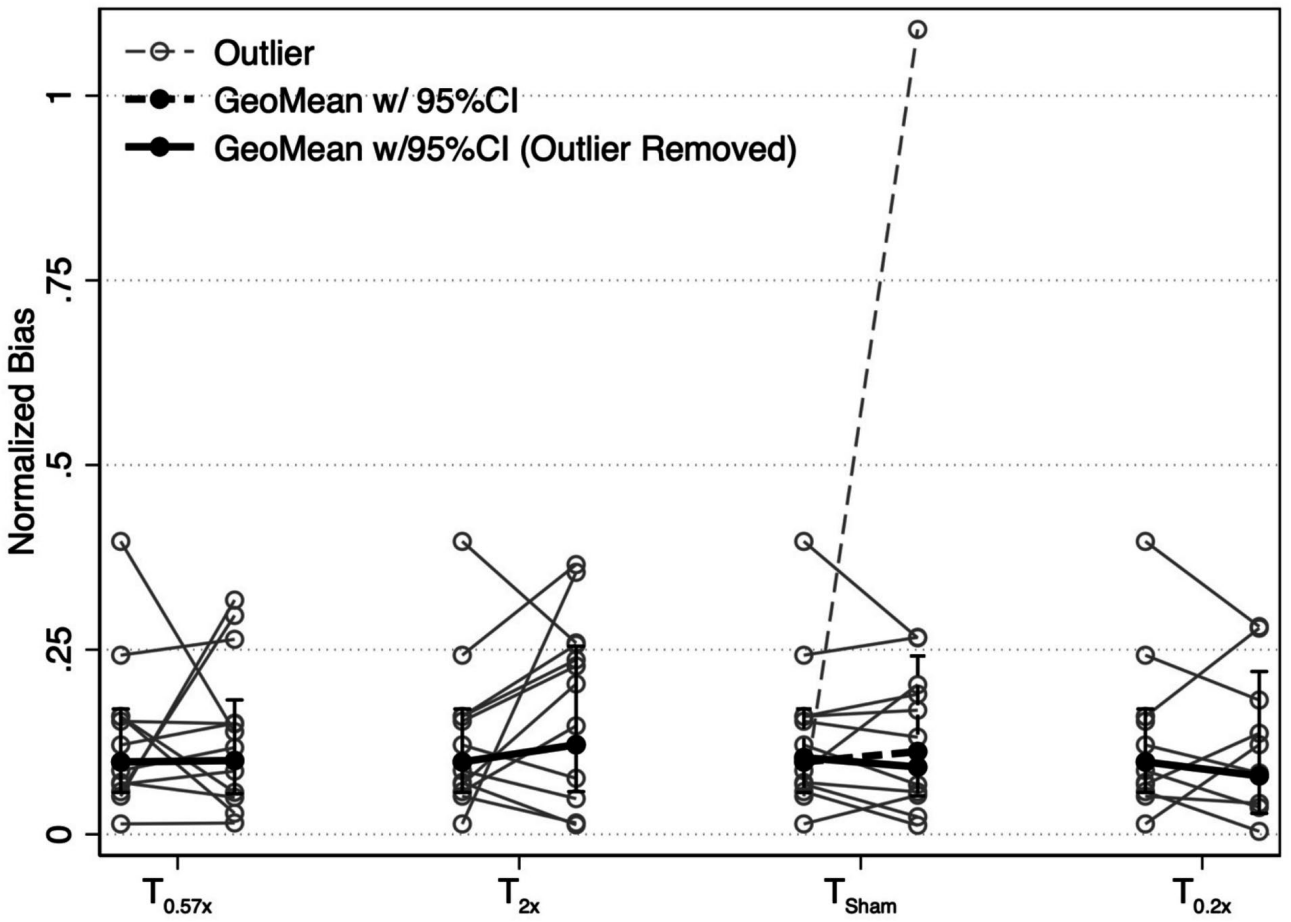
Changes in the absolute value of the normalized bias parameter are shown for each conditioning task. Solid black lines show the geometric mean of the bias, with error bars representing the 95% confidence interval. One participant showed an exaggerated bias after the T_Sham_ condition (narrow dashed line). Results with this participant included (wide dashed line) and removed (wide solid line) are shown. Removal of this individual did not change our findings. T_0.57x_, 57.4% of threshold; T_2x_, 200% of threshold; T_Sham_, 1 mm/s peak velocity 1 Hz interaural translations; T_0.2x_, 20% of threshold.

After the T_2x_ conditioning block, yaw rotation thresholds (1.36 ± 0.75°/s) were significantly increased compared to baseline (*β* = 0.303, *p* = 0.00414, Figure 2), increasing an average of 31.75 ± 38.71% (−24.64 to +100.18%, Figure 3). The change in thresholds relative to baseline was also significantly greater than the change observed following the sham condition (*p* = 0.012). A paired t-test with one-sided *p*-value also showed a significant increase in the post-condition threshold compared to baseline (*t*(11) = 2.56, *p* = 0.013). These data suggest that yaw rotation thresholds are significantly increased after suprathreshold motion, and to a greater extent than after a sham stimulus. At the individual participant level, two individuals showed a decrease in thresholds by 20.82 and 24.64%, two individuals showed a nominal change between-7.52 and + 7.82%, and the remaining 8 showed an increase of greater than 22% after the T_2x_ condition (Figure 2). Normalized bias was not significantly different from baseline (*β* = 0.052, *p* = 0.46, Figure 4).

**Figure 5:**
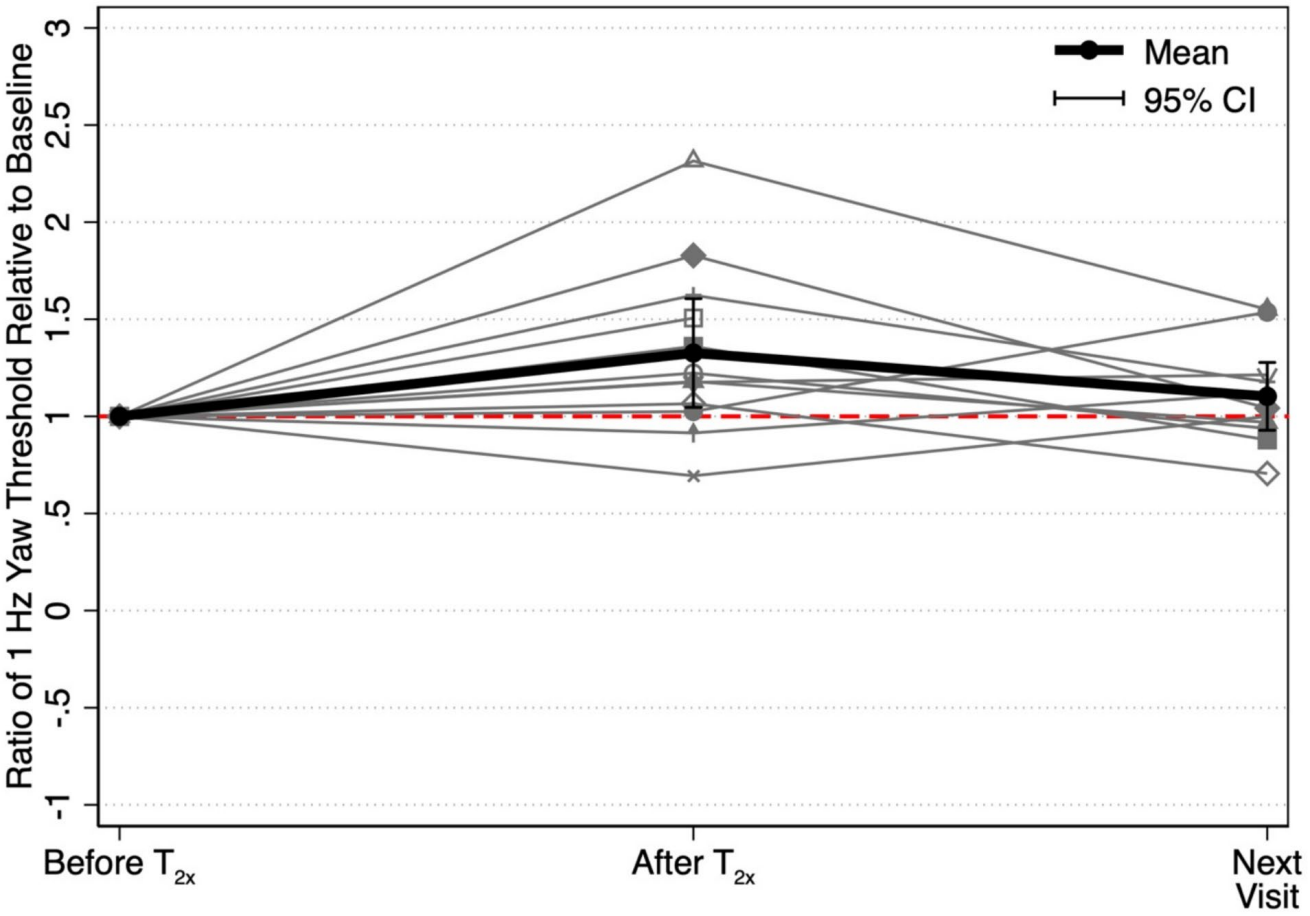
Ratios between each threshold and the baseline threshold captured prior to the T_2x_ conditioning stimulus (“Before T_2x_”) are shown to depict the relative to change in yaw rotation thresholds at each of two time points, immediately following conditioning (“After T_2x_”) and at the next visit to the laboratory (“Next Visit”) (i.e., 1.0 = equivalent to T_2x_ baseline). Mean threshold ratios and the corresponding 95% confidence intervals are shown at each time point. Yaw rotation thresholds were estimated from a single block of 150 trials.

We were able to collect only a single post-conditioning assessment, and as a result, are unable to determine a precise duration for the observed increase in perceptual thresholds. However, 11 of the 12 participants did return to the lab for an additional visit after completing the T_2x_ visit (median interval = 7 days, range = 1 to 88 days). We found that thresholds measured at the subsequent visit to the laboratory (1.16 ± 0.48°/s) were not significantly different from thresholds assessed prior to the suprathreshold conditioning block (1.07 ± 0.60°/s, *t*(10) = −0.52, *p* = 0.4865) (Figure 5).

### Effect of T_0.57x_ subthreshold conditioning in healthy controls

Yaw rotation thresholds measured after the T_0.57x_ conditioning stimulus (1.11 ± 0.62°/s) were not significantly different from baseline (*β* = 0.0504, *p* = 0.612) (Figure 2). The absence of a significant reduction in thresholds after conditioning was further supported by the results of a paired t-test with one-sided *p*-value (*t*(11) = 0.69, *p* = 0.75). On average, thresholds were increased by 4.59 ± 23.28% (Figure 3). For 11/12 participants, thresholds were within ±25% of baseline, however one participant showed a 64.39% increase in their yaw rotation threshold. The estimated difference in thresholds relative to baseline was similar for the T_0.57x_ condition and the sham condition (*p* = 0.951). The normalized bias also was not significantly different from baseline (*β* = 0.0075, *p* = 0.914, Figure 4). Counter to our hypothesis, these data support that yaw rotation thresholds were not significantly decreased after subthreshold conditioning.

### Effect of T_0.2x_ subthreshold conditioning in healthy controls

Similar to the T_0.57x_ condition, yaw rotation thresholds (1.20 ± 0.69°/s) did not differ significantly from baseline (*β* = 0.106, *p* = 0.385, *N* = 9) after the lower velocity T_0.2x_ condition, increasing an average of 6.61 ± 26.3% (*t*(8) = 1.25, *p* = 0.88, Figures 2, 3). Moreover, when analyzed separately for other conditioning stimuli, this subset of the HC sample performed similarly to the entire cohort, with no differences in thresholds after the T_0.57x_ (*β* = 0.089, *p* = 0.462) or T_Sham_ (*β* = 0.071, *p* = 0.559) conditions, and a significant increase in thresholds after T_2x_ conditioning (*β* = 0.386, *p* = 0.00296). Normalized bias was also not significantly different (*β* = −0.0035, *p* = 0.967, *N* = 9, Figure 4).

### Subthreshold conditioning in participants with vestibular loss

**Figure 6:**
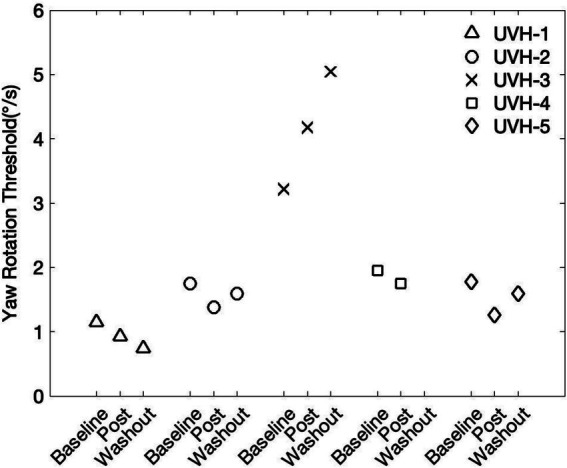
Five participants with a history of chronic unilateral vestibular hypofunction (UVH) piloted the T_0.57x_ subthreshold conditioning task. In four out of the five participants, yaw rotations thresholds were at least 10% lower at the post-conditioning assessment. The washout period consisted of a 30-min break outside the testing room.

In control conditions, making small changes could be challenging due to homeostatic plasticity, which counteracts imposed changes. We therefore used the T_0.57x_ conditioning in patients with UVH to determine if it could improve their thresholds. Indeed, in the cohort of individuals with chronic UVH, 4 out of the 5 participants showed a decrease in 1 Hz yaw rotation thresholds after the T_0.57x_ conditioning stimulus (Table 2 and Figure 6). The magnitude of change ranged from 10.32 to 29.14% decrease (Median = 18.91%, interquartile = 10.32, 20.72). One participant (UVH-3) showed a 64.73% increase in thresholds at the post-conditioning assessment. None of the 5 participants with UVH reported adverse sensations during, or after, the block of subthreshold conditioning. In addition, when prompted, participants reported an inability to sense the direction of motion during the conditioning block.

In 4 of the 5 participants, a 2^nd^ follow-up threshold assessment was able to be captured after a 30-min washout period. In two of the individuals who showed an immediate reduction in thresholds, yaw rotation thresholds increased back toward baseline (UVH-2 and UVH-5, Figure 6). In one individual, thresholds were further lowered away from baseline (UVH-1), and in the one participant with elevated thresholds after conditioning (UVH-3), thresholds continued to increase after the washout period (Figure 6). In the individual with elevated thresholds, the changes were transient. The yaw rotation threshold estimated from the initial block of 100 trials (2.72 deg/s) performed prior to T_0.57x_ conditioning was similar to thresholds estimated from an additional 200 trials captured approximately 1 month later (2.51 deg/s).

### Correlation between baseline thresholds and response to conditioning stimuli

**Figure 7:**
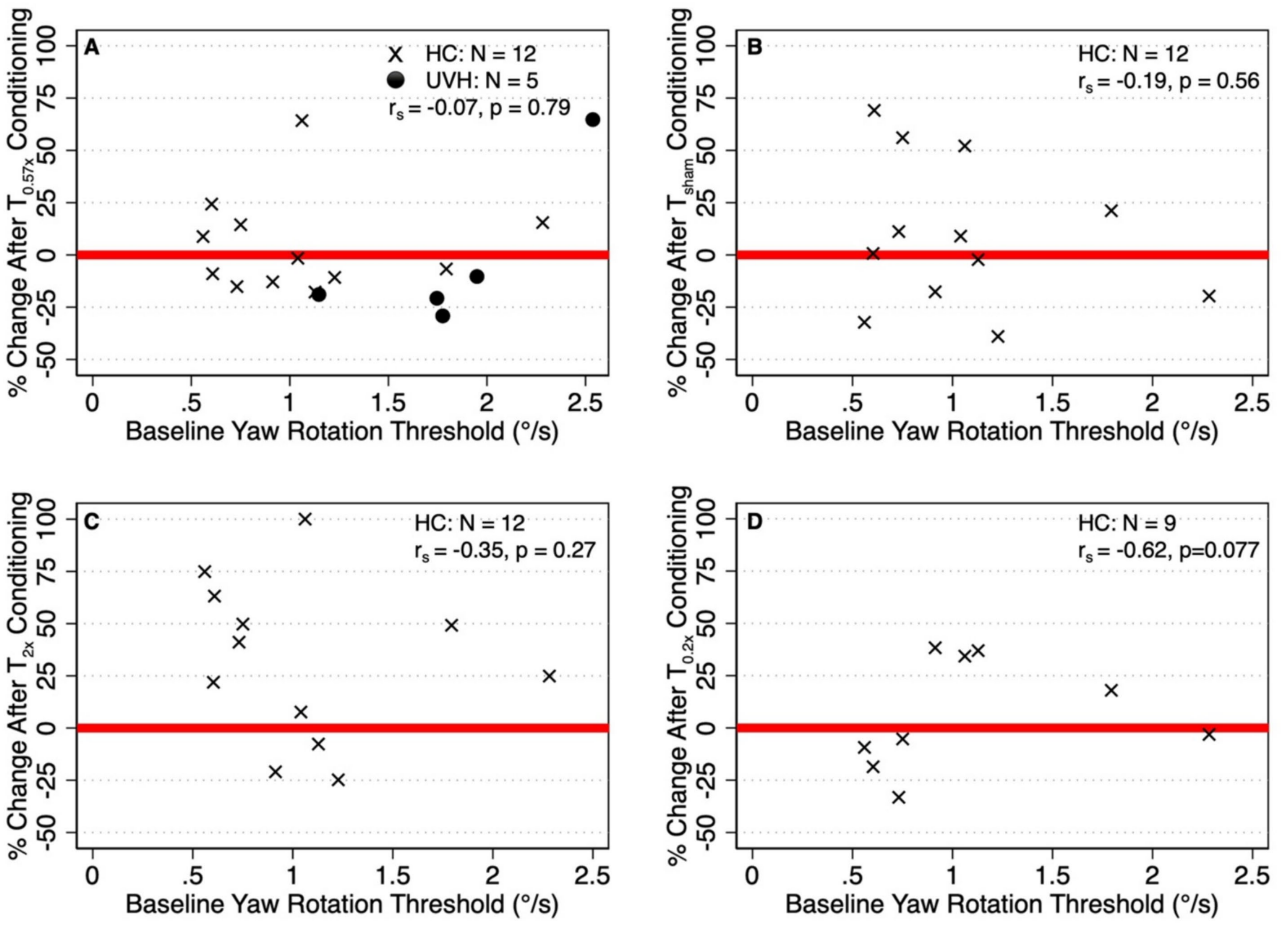
**(A)** Relationship between the percent change in thresholds after T_0.57x_ conditioning and baseline thresholds in both the healthy control cohort (HC, *N* = 12, x) and unilateral vestibular hypofunction (UVH, *N* = 5, ⚫) cohorts. **(B–D)** Show the correlations between baseline thresholds and the change in thresholds after the T_sham_ T_2x_, and T_0.2x_ conditions, respectively, in the HC cohort. Red lines placed at *y* = 0 are shown to differentiate between participants whose thresholds were increased (i.e., percent change greater than 0) and decreased (percent change less than 0) relative to baseline.

To test any possible effect of initial conditions, we investigated whether the baseline thresholds were related to those after conditioning. Across both cohorts (*N* = 17), baseline perceptual thresholds were not significantly correlated with the percent change in thresholds after the T_0.57x_ condition (*r_s_* = −0.07, *p* = 0.79) (Figure 7A). Similarly, baseline thresholds were not significantly correlated with the percent change in thresholds after the T*_sham_* (*r_s_* = −0.19, *p* = 0.56, Figure 7B) or T_2x_ (*r_s_* = −0.35, *p* = 0.27, Figure 7C) conditions in the HC cohort (*N* = 12). In the subset of the 9 HC participants who returned for the additional day of testing, we found a moderate, although not significant, negative correlation between baseline thresholds and the percent change in thresholds after the T_0.2x_ condition (*r_s_* = 0.62, *p* = 0.077, Figure 7D).

## Discussion

We found that a 20 min block of 1 Hz suprathreshold sinusoidal yaw rotations led to a significant increase in perceptual thresholds in HC participants and that the average change surpassed the anticipated within-subject variability of a 1σ threshold estimate (SD of ~13.2% after 150 trials, SD of ~7.83% after 450 trials) (35, 37). Yet, in contrast to our hypothesis of bidirectional modulation of yaw rotation perception, yaw rotation thresholds were not significantly reduced after a subthreshold conditioning stimulus in our HC cohort. However, in a cohort of 5 individuals with chronic UVH, we did find evidence of reduced thresholds following the same subthreshold yaw rotation stimulus (T_0.57x_). Based on these findings, we suggest that suprathreshold motion provides a sufficient stimulus for decreasing sensitivity to yaw rotation cues in healthy adults, whereas subthreshold conditioning may be more salient for individuals with a history of vestibular loss.

Our data showing a reduction in sensitivity after suprathreshold rotation is consistent with previous animal and human studies. In *Xenopus laevis*, Dietrich and Straka (8) found a 27% reduction in the firing rates of oculomotor neurons in the VOR pathway after 20 min of 60°/s sinusoidal yaw rotations (i.e., 2x the 30 deg/s baseline condition). This effect was also present in the absence of visual feedback, supporting that plasticity in the VOR pathway occurred independent of the standard visual-vestibular error signal (i.e., retinal slip) (8). In a sample of healthy adults, Fitzpatrick and Watson (11) found that 10 min of stochastic 100°/s yaw rotations led to a 248% reduction in the perceived size of whole-body yaw rotation and an attenuation of balance and perceptual responses to a galvanic vestibular stimulus. Dietrich and Straka attributed these collective findings to a homeostatic response to the prolonged change in neural activity induced by the higher velocity yaw rotation stimulus, with the lowered neural excitability acting to stabilize the subsequent encoding of vestibular signals (8). Two lines of evidence suggest that the observed increase in perceptual thresholds following T_2x_ suprathreshold conditioning in this study are also due to homeostatic changes rather than habituation. First, previous studies have shown similar changes with suprathreshold stimuli, but also have shown a change in the opposite direction with subthreshold stimuli (8). Second, habituation effects are shown after step stimuli and last for weeks to months after a single trial, which is in contrast to shorter lasting effects of sinusoidal stimuli in the range of minutes, suggesting a homeostatic mechanism. For example, repeated velocity steps about an earth vertical axis have been found to cause a habituation to subsequent rotations, characterized by a reduction in VOR gain, an increase in phase lead, and an attenuation of rotation perception (40–43), with the changes lasting weeks to months after a single exposure. In contrast, Clement et al. found only a weak and transient change in VOR responses to sinusoidal rotations, with the effects being most noticeable at the lowest frequencies tested (0.02 and 0.04 Hz) (44). In human subjects, Baloh similarly found only a transient increase in VOR phase lead after low frequency (0.005–0.01 Hz) sinusoidal accelerations, with inconsistent carry-over of the changes to subsequent visits (40). In their study of *Xenopus laevis*, Dietrich and Straka found that 20 min of 0.5 Hz sinusoidal yaw rotations at 30 deg/s had no effect on firing rates during subsequent sinusoidal rotations performed at the same frequency/velocity (8). The pronounced effects of habituation on the vestibular response to velocity steps, but not to sinusoidal rotations, suggests that the nature of the motion stimulus may influence the nature of the adaptive response. Specifically, habituation may serve primarily to suppress the vestibular response to stimuli that are dissimilar to or out of the range of naturalistic motion—such as those experienced during unidirectional steps of angular velocity and low frequency sinusoidal rotations (44). Homeostatic responses may instead serve to regulate sensitivity to motion stimuli in the range of head movements typically experienced during naturalistic motion, including the 1 Hz stimulus used here (45), as well as the 0.5–2.5 Hz stimuli used in past studies of vestibular conditioning 0.5–2.5 Hz (8–11). Therefore, we suggest that the observed increase in thresholds after suprathreshold conditioning likely represents a homeostatic response, as opposed to habituation.

Our results showing perceptual threshold changes only in one direction are in contrast with those of two other studies. First, Dietrich and Straka found that firing rates of oculomotor neurons during a 30°/s test condition were increased 25% after 20 min of 6°/s yaw rotations (not subthreshold) (8), and decreased 27% after 60°/s yaw rotations (as described above). The lack of an effect of subthreshold conditioning on perceptual thresholds in our healthy control cohort contrasts with these findings. Since the stimulus used in their study was 20% of the reference condition (6°/s vs. 30°/s), we hypothesized that our lack of an effect may have stemmed from excessive velocity of the T_0.57x_ subthreshold stimulus (57.4% of baseline). To test this hypothesis, we brought back 9 participants to complete a second T_0.2x_ conditioning stimulus that used yaw rotations with a peak velocity equal to 20% of the baseline threshold. However, we again found insufficient evidence to reject the null hypothesis that thresholds were similar before and after subthreshold conditioning. Outside of species-specific differences, a possible explanation for this finding is that subthreshold yaw conditioning might have disparate effects on self-motion perception compared to the VOR, and/or might preferentially influence unique frequencies of yaw rotation perception outside of the 1 Hz stimulus tested here. Alternatively, differences may have resulted from our use of a subthreshold, as compared to a suprathreshold, conditioning stimulus. The ability to increase yaw rotation thresholds with a large suprathreshold yaw rotation stimulus, as shown here and previously by others (11), highlights the potential regulatory effects of suprathreshold motion. Future work should determine if weak suprathreshold yaw rotations—performed at a velocity above threshold but below the T_2x_ stimulus used here—might enhance the perception of subsequent yaw rotation cues.

Second, Keywan and colleagues found a 28.8% reduction in interaural (IA) translation vestibular perceptual thresholds after a 20 min subthreshold conditioning stimulus (70% of the 0.82σ threshold, i.e., 57.4% of 1σ threshold) (9, 10). The conflicting effects of subthreshold conditioning on self-motion perception between our two studies suggests a difference in the relative modifiability of the perception of gravitoinertial acceleration signals from the otoliths compared to angular velocity signals from semicircular canals, which could be due to differences in the way the sensors encode linear and rotational movements. Such differences arise at the level of hair cell stimulation mechanisms (46, 47) and result in different response patterns and information transmission by canal and otolith afferents (12, 46, 47). Note, it is unlikely that the absence of an effect in our study was due to a failure to displace the endolymphatic fluid, since subthreshold conditioning stimuli had accelerations of greater than 0.3°/s^2^ for each of the participants and an acceleration of 0.1°/s^2^ effectively deflects the cupula (48). Studies of vestibular perceptual training in healthy controls (49, 50) have similarly shown differences in the modifiability of thresholds depending on the nature of the motion stimulus, with more complex motions that require canal-otolith integration (i.e., roll tilt) being more susceptible to training than IA translation or yaw rotation (51).

The effectiveness of our subthreshold conditioning stimulus to affect the canals was further shown by the change in perceptive thresholds in UVH subjects. Four out of the 5 UVH subjects showed lower thresholds after 20 min of subthreshold T_0.57x_ conditioning. This change was unlikely to be the result of a regression to the mean, since baseline thresholds did not predict the degree of change after conditioning. It is possible that such homeostatic responses in UVH subjects may have resulted from changes in the ipsilesional vestibular nuclei (12, 52). After UVH, the loss of ipsilesional afferent inputs triggers a cascade of compensatory changes, including increased sensitivity at the ipsilesional vestibular nuclei (VN) synapses (12, 17, 53, 54). Interestingly, homeostatic plasticity is believed to alter neuronal firing rates through a similar mechanism, by dynamically scaling synaptic weights (1, 7). Thus, unlike in healthy adults who have little need to further lower yaw rotation thresholds, subthreshold conditioning may preferentially impact perceptual thresholds in individuals who have yet to achieve a stable perceptual performance level due to incomplete compensation. Alternatively, young healthy adults may require a longer duration conditioning stimulus in order to modify yaw rotation thresholds. Mechanistic questions should be addressed in future studies, including the potential influences of alternative pathways such as efferent-mediated changes in vestibular afferents (54, 55).

### Limitations

As in previous studies on threshold changes, interpretation of the results of our study should take into account the small sample size. In addition, the observed changes in thresholds should be considered relative to the anticipated within-subject variability of perceptual threshold estimates. In this study we found that the average change in thresholds after suprathreshold conditioning was more than twice the expected within-subject variability for a threshold estimated from 150 trials of a symmetric 4D/1 U staircase (35, 37). Since perceptual threshold protocols requires a balance between increased precision and prolonged test duration (i.e., efficiency), achieving more precise threshold estimates came at the expense of a prolonged post-test assessment. As a result, changes in perceptual performance may have changed over the course of the 10-min threshold assessment. Finally, although the duration of test sessions were comparable for all subjects, the long assessment sessions could have resulted in fatigue and/or inattention, influencing perceptual thresholds differently in different individuals. However, we anticipate that such effects would be similar for the same individual across different test conditions. Additional work is needed to define test protocols that are sufficiently precise and rapid enough to allow for incremental tracking of changes after vestibular stimulation protocols.

## Data Availability

The raw data supporting the conclusions of this article will be made available by the authors, without undue reservation.
